# Climate Change and Children’s Health: A Commentary

**DOI:** 10.3390/children2040412

**Published:** 2015-10-15

**Authors:** Fiona Stanley, Brad Farrant

**Affiliations:** The Telethon Kids Institute, The University of Western Australia, P.O. Box 855, West Perth 6872, Australia; E-Mail: Brad.Farrant@telethonkids.org.au

**Keywords:** climate change, child health and wellbeing, global warming, wicked problems, capitalism

## Abstract

This commentary describes the likely impacts on children's health and wellbeing from climate change, based on the solid science of environmental child health. It describes likely climate change scenarios, why children are more vulnerable than older people to these changes, and what to expect in terms of diseases (e.g., infections, asthma) and problems (e.g., malnutrition, mental illness). The common antecedents of climate change and other detrimental changes to our society mean that in combatting them (such as excessive consumption and greed), we may not only reduce the harmful effects of climate change but also work towards a better society overall—one that values its children and their futures.

## 1. Science and Its Limitations

Man-made climate change is occurring, it is a major challenge for the 21st century, and it is already affecting the health and wellbeing of populations. Any scientific evidence, particularly for something as complicated as the response of the planet and all its systems, can never perfectly predict trends nor tell us precisely what to do. In public health, we face this all the time: the human body is also a very complex organism with huge variability in both genetic influences and environmental exposures from conception and over the lifespan. We make public health policy which often targets large numbers of people based on imperfect science, but of course we continue to monitor and research the problems and adapt policies and practices as new evidence becomes available.

**Figure 1:**
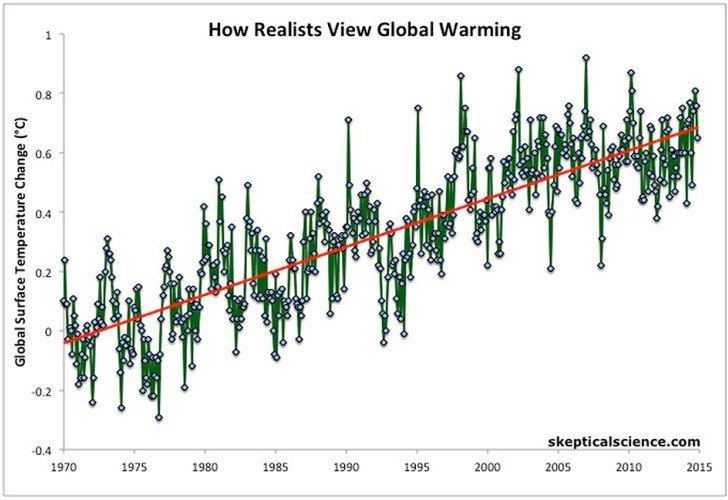
Global surface temperature change (from Skeptical Science [56]).

However, surely intelligent citizens can observe the data that is currently available and not contestable. Globally, 2014 was the hottest since records began and was the 38th consecutive year that was above the average [1]. It is foreseen that 2015 will be even hotter, with January through July having the hottest combined land and ocean temperatures on record and July setting a new high for the hottest month since records began in 1880 [2]. In Australia, 2013 was the hottest on record with 2014 the third hottest on record [3]. Despite claims that we have seen a pause, averaging out temperature over the last 45 years shows a steady increase (see Figure 1). Rainfall has been decreasing in South and Western Australia [4], and CO_2_ emissions are continuing to rise [5]—overwhelmingly due to fossil fuel burning [6]. In March 2015, global atmospheric carbon dioxide concentrations were above 400 parts per million for a whole month for the first time since records began [7]. This is a rise of more than 120 parts per million since pre-industrial times and is well above the ‘safe’ level of 350 parts per million [8].

The Organisation for Economic Co-operation and Development (OECD) has recently released its predictions for the world economy to 2060 [9]. In summary these are that growth will slow to around two thirds its current rate, that inequality will increase massively and that there is a big risk that climate change will make things worse. To quote Paul Mason (Guardian Weekly 18–24 July 2014) *“The whole projection is overlaid by the risk that the economic effects of climate change begin to destroy capital, coastal land and agriculture in the first half of the century, shaving up to 2.5% off world GDP (gross domestic product) and 6% in southeast Asia”.* The message from the OECD is important for rich countries—the best of capitalism is now over. We would like to suggest that climate change is but one outcome of a series of serious and wicked problems that are in major part due to rampant capitalism. This commentary will describe how an epidemiologist interested in child health approaches the wicked problems affecting our society, touch on the known likely effects of the wicked problem of climate change on children’s health and then illustrate the negative effects of a world that focuses excessively on wealth creation. Maybe the behaviour changes required to reduce the impact of climate change can also create a more civil society.

## 2. Wicked Problems Facing Society

Business often uses the concept of “wicked problems” but it is also a useful frame for many of the threats to public health [10,11,12,13,14,15]. The term wicked problems was coined in 1973 by Rittel and Webber to refer to problems that are tough to describe, have multiple causes and don’t have a single right answer [16]. More recent conceptions underscore the uniqueness, complexity and enigma of wicked problems, highlight the fact that they can’t be solved by the standard approaches that have been used in the past, and point out that wicked problems are often the symptoms of other underlying problems [10]. The characteristics of wicked problems mean that they are difficult to address by any one agency or bureaucracy and have major societal effects.

**Figure 2:**
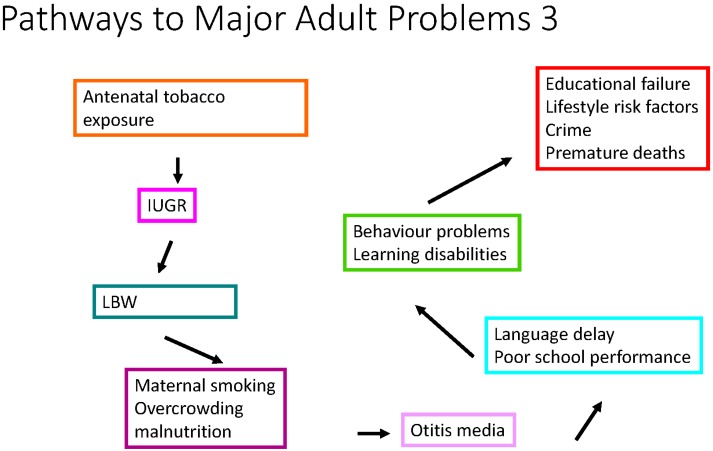
Pathways to major adult problems. IUGR: intrauterine growth restriction. LBW: low birth weight.

Examples of wicked problems affecting children and young people include complex conditions such as obesity, mental ill health, substance abuse, child maltreatment, behavioural and educational problems, juvenile crime and the broader societal ones—climate change, environmental degradation, and inequalities (socio-economic, in health, education and opportunity). The pathways from antenatal tobacco or alcohol exposure (see Figure 2) demonstrate both the complexity of the range of poor outcomes in children of mothers who have these pregnancy exposures and also the challenges in preventing or ameliorating their effects. These wicked “health” problems are not going to be solved by health departments but by joined up interventions which appreciate the complex antecedents and circumstances that result in them occurring. Thus wicked problems demand both a whole of government and a whole of society approach. Climate change is one of these wicked problems and its impacts on children’s health and wellbeing are likely to be profound.

## 3. Climate Change and Child Health

The innocent and non-consenting victims of climate change, the children of today and tomorrow, are also particularly vulnerable to its negative impacts. According to recent estimates, children already suffer around 90% of the global disease burden from climate change [17] with almost all of this occurring in developing countries which are the least responsible for climate change [18,19]. Globally, for children under 5 years of age climate change is predicted to worsen all of the top five causes of death (malnutrition, neonatal deaths, acute respiratory illness, diarrhoea, malaria) [19].

Even though the protection of children should be society’s highest calling there is the very real prospect that lack of action to mitigate and adapt to climate change will mean that our children and future generations will be the first to have poorer health and mental health than the generation before them. Indeed, the lag between cause and consequence makes climate change a major intergenerational health equity issue [20]. One of the world’s leading climate scientists, James Hansen, has long argued that the adults of today have a moral responsibility to current and future generations of children to do what is required to prevent dangerous global warming:

*“Exposure to media ensures that children cannot escape hearing that their future and that of other species is at stake, and that the window of opportunity to avoid dramatic climate impacts is closing. The psychological health of our children is a priority, but denial of the truth exposes our children to even greater risk”*.

[21]

Climate change will affect child health around the world with different consequences in different regions and population groups [22,23] yet the recent Intergovernmental Panel on Climate Change Working Group 2 report emphasised a lack of preparedness for climate change impacts in many countries [24]. Although more research is required at the regional and local levels in order to better understand, prepare for and adapt, the health and other challenges posed by climate change are becoming increasingly clear. Those living near rivers (more frequent and intense floods), seasonally arid areas (increased drought and bushfires) and areas prone to water scarcity are likely to be most affected [22]. Climate change has and is expected to further amplify the social gradient in health leading to even greater health inequity because the people who are, and will continue to be, among the first and most affected are the poor and disadvantaged who have higher disease burdens and less resources with which to protect themselves [20,23]. Globally, the negative effects of climate change on agricultural productivity and food security are predicted to cause a 20% increase in the number of malnourished children by 2050 [25]. It has also been predicted to cause as many as 200 million additional ‘environmental refugees’ by 2050 [26] with this forced migration having adverse mental, physical and social health impacts on children [27].

**Figure 3:**
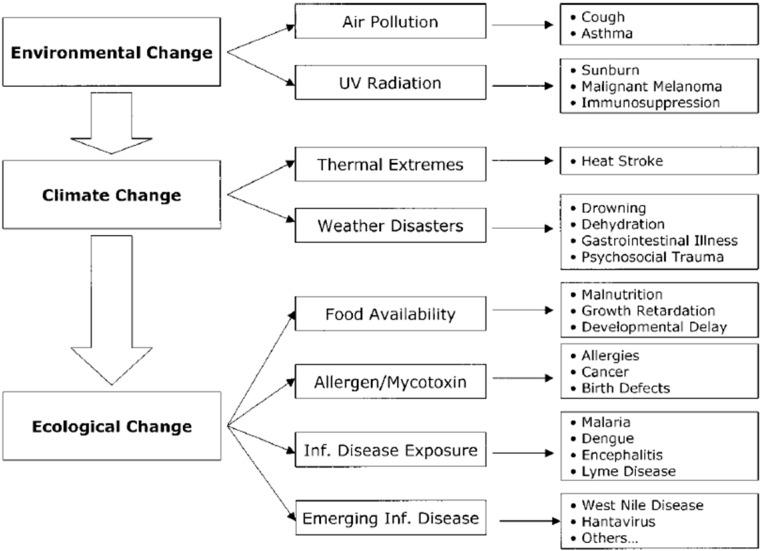
The relationship between environmental change, climate change, ecologic change, and child health [28].

The changes in our living conditions which are accompanying climate change, environmental and ecological change and how they are affecting child health are shown in Figure 3 [28]. The increases in air pollution increase respiratory conditions [29] and thermal extremes and weather disasters can result in a range of diseases and complications from heat stroke through to psychosocial trauma [20,30,31,32,33]. The ecological changes brought about by climate change are likely to manifest their effects through decreases in the availability of food and clean water causing malnutrition, diarrhoea, poor growth and developmental delays in early childhood and increases in allergens causing allergies and asthma [22,25,28,34,35]. Such outcomes are already on the increase in areas of China which are also suffering high levels of environmental pollution due to high levels of smoke from fossil fuels in the atmosphere in large urban areas. This highlights the fact that mitigating climate change by reducing fossil fuel use has both direct (via reduced pollution, *etc.*) and indirect (via reduced harm from climate change) benefits for child health. Changing temperatures and environments are also resulting in increases in infectious diseases with some infections which only occurred in tropical areas moving into more temperate zones and also new emerging infections appear on the increase [36,37].

**Figure 4:**
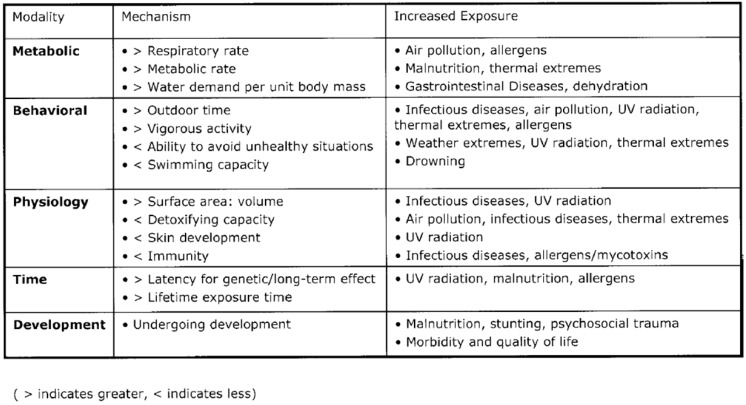
Modalities and mechanisms by which children may be more susceptible to climate change than adults [28].

Figure 4 suggests the reasons why children are especially vulnerable to climate change and associated environmental situations [28]. They are smaller, have immature development and immune systems, have higher metabolic and respiratory rates, and a higher intake per unit body mass. They are likely to spend more time outdoors in vigorous activity which will increase exposures and are less capable of avoiding unhealthy situations. Their ability to handle infections, toxins and other exposures is limited.

Children are also particularly vulnerable to toxic levels of stress because of their immature neurobiology [38]. Research has found higher rates of psychological distress and emotional problems as well as elevated symptoms of Post-Traumatic Stress Disorder in children after climate related “natural” disasters [39,40]. There is also evidence that children’s mental health is already being diminished by anxiety about future climate change [37]. Increased exposure to climate induced extreme weather events and related disasters means that children will be exposed to elevated levels of trauma and stress in-utero and during childhood and this will likely result in marked changes in brain development and longer term cognitive and mental health impacts [20].

As they are young now their lifetime of exposure to the damaging effects of climate change is going to be high. Children are more vulnerable in so many ways than adults to the variety of problems posed by climate change [41]. Whilst the science of the effects of climate change on children’s health is still limited and it is “early days”, these aspects summarised here are all based on the solid science of environmental child health and these predictions are based on the best evidence from that area and are likely to be accurate.

## 4. Towards a More Civil Society

*“We are witnesses to a dramatic expansion of market-based economies whose capacity for wealth generation is awesome in comparison to both the distant and the recent past. At the same time, there is a growing perception of substantial threats to the health and wellbeing of today’s children and youth in the very societies that benefit most from this abundance”*.

[42]

There is no doubt that there has been a major increase in financial wellbeing over the last 100 years and that capitalism has contributed to the improved situations of millions of families world-wide. However over the last 20–30 years, as capitalism has become more extreme and focused solely on wealth creation, we have observed a range of dramatically increasing wicked problems affecting children and youth in the wealthiest countries. Suicide rates and mental health problems in young people quadrupled in Australia from 1980 to the 2000s; behavioural problems and learning disabilities have dramatically increased along with asthma and obesity. Many of the wealthiest countries have the highest inequalities across their societies which are extremely detrimental to family, child and youth health and wellbeing. A powerful warning about the pitfalls of an over-emphasis on material wealth as a measure of progress was issued nearly half a century ago:

*“Yet the gross national product does not allow for the health of our children, the quality of their education, or the joy of their play. It does not include the beauty of our poetry or the strength of our marriages; the intelligence of our public debate or the integrity of our public officials. It measures neither our wit nor our courage; neither our wisdom nor our learning; neither our compassion nor our devotion to our country; it measures everything, in short, except that which makes life worthwhile. And it tells us everything about America except why we are proud that we are Americans.”*.

Robert F. Kennedy Address, University of Kansas, Lawrence, Kansas, March 18, 1968 [43]

**Figure 5:**
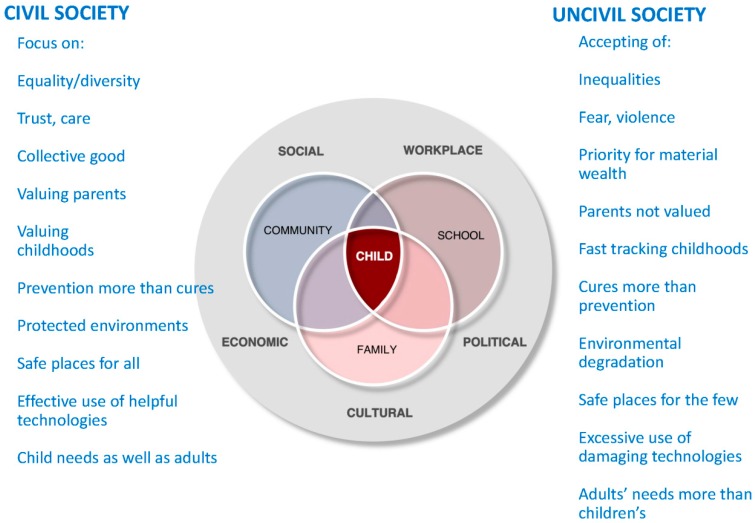
Ecological factors in Child Wellbeing (Stanley).

Figure 5 describes those aspects of a civil society which enables children to grow up healthily and strong within their families and communities and points out that the major enablers or disablers are well outside the family and community units—they are in the broader social, economic, political, cultural and workplace values and environments that we as a society create. And in the second decade of the 21st century, the enablers are being overcome by the disablers.

Because of the particular vulnerability of children and the way that climate change will amplify existing inequities in burden of disease and other child outcomes it is not possible to adequately protect and improve child health without addressing and overcoming the factors that produce the existing social and economic gradients in child health outcomes [23]. A growing number of authors from a wide range of fields are pointing out the massive negative climate, health and other environmental impacts of predatory capitalism-consumerism where people have become dehumanised objects in service of the corporate bottom line and where the guiding ethical principle is the maximisation of shareholder profits (regardless of the human and environmental costs). In his best-selling book, award winning economist Thomas Piketty argues that inequality and the increasing concentration of wealth in the hands of a few (and the associated negative outcomes) are features of capitalism that require a major overhaul including an annual global wealth tax and progressive income taxes of as high as 80% [44]. Best-selling author, social activist and filmmaker Naomi Klein goes further in her book *This Changes Everything: Capitalism vs. the Climate* arguing that, by blocking the reforms that are needed to address climate change, capitalism is waging a war against life on earth and that to prevent the addiction to profit and growth from taking us closer to dangerous global warming every day requires us to break all the “free-market” rules by reigning in corporate power, reclaiming our democracies and rebuilding local economies [45]. Others, including health researchers like Dr. Tony Waterston and Dr. Arnab Seal, advocate sustainable development where everyone (consumers, corporate and government leaders, *etc.*) is individually and collectively responsible for ensuring that improvements in their quality of life do not come at the expense of the needs of others now or in the future [41,46].

Healthcare settings have large carbon footprints and have been urged to lead by example in making large reductions and professionals involved in the health sector have also been urged to use their position to influence the public and policy debates on the need for urgent greenhouse gas emission reductions to prevent the negative health impacts associated with dangerous global warming [19,46,47,48,49,50]. Resources and recommendations to assist the health sector have been produced by the World Health Organisation, Health Care Without Harm, International Society of Doctors for the Environment and the Climate and Health Council [51,52,53,54].

## 5. Conclusions

While we don’t profess to have all the answers, we believe there is growing evidence to support the argument that the factors driving climate change are the same as those driving corporate greed, excessive individual wealth creation, inequalities and all the other wicked problems our society faces. Hence if we can better understand and change these underlying factors we may not only reduce the impact of climate change on children and young people, but our whole society will be a better place for all. Our intention here is to help raise awareness of these issues and to urge others in the research and health sectors to get involved in the conversations and actions aimed at protecting and bettering the futures of the children of today and tomorrow.

*“In the long run, the harm to human health from climate change is more than an avoidable burden of suffering, injury, illness and premature death. It signals that our mismanagement of the world’s climate and environment is weakening the foundations of health and longevity”*.

[55]
